# Accuracy of motor assessment in the diagnosis of fetal alcohol spectrum disorder

**DOI:** 10.1186/s12887-019-1542-3

**Published:** 2019-05-28

**Authors:** Danielle Johnston, Erin Branton, Leah Rasmuson, Sylvia Schell, Douglas P. Gross, Lesley Pritchard-Wiart

**Affiliations:** 1 Alberta Health Services, Central Zone East, Children’s Rehabilitation Services, Professional Centre, Suite 300, 5015 50 Ave, Camrose, Alberta T4V 3P7 Canada; 2 Department of Physical Therapy, Faculty of Rehabilitation Medicine, University of Alberta, 2-50 Corbett Hall, Edmonton, Alberta T6G 2G4 Canada

**Keywords:** Fetal alcohol spectrum disorder (FASD), Fetal alcohol syndrome (FAS), Prenatal alcohol exposure (PAE), Motor skills, Gross motor, Fine motor, Assessment, Child and youth development, Diagnosis, Accuracy

## Abstract

**Background:**

To evaluate the accuracy of motor assessment tools listed in *Fetal alcohol spectrum disorder: a guideline for diagnosis across the lifespan* (Canadian Guideline) for the purpose of fetal alcohol spectrum disorder (FASD) diagnosis. Specifically, we aimed to determine: 1) diagnostic accuracy of motor assessment tools and subtests; 2) accuracy of multiple subtests versus total scores; and 3) accuracy of alternate cut-offs.

**Methods:**

Cross-sectional diagnostic study of 63 children aged 6–17 years. Diagnostic accuracy and alternate cut-offs were calculated for the Movement Assessment Battery for Children, 2nd edition (MABC-2), Bruininks-Oseretsky Test of Motor Proficiency, 2nd edition Short Form (BOT-2SF) and Beery-Buktenica Developmental Test of Visual Motor Integration, 6th edition (BeeryVMI-6).

**Results:**

The MABC-2 total motor score was more sensitive (0.30; 95% CI 0.17–0.46; *p* < 0.01) to motor impairment in the presence of FASD than the BOT-2SF (0.02; 95% CI 0.00–0.12) at the 2nd percentile (−2SD). The MABC-2 total motor score was more accurate than any combination of subtest scores. The Motor Coordination subtest of the BeeryVMI-6 (BeeryMC) at the 5th percentile (− 1.5SD) (sensitivity 0.68, specificity 0.90) was the most accurate subtest.

**Conclusions:**

The BOT-2SF was an inaccurate assessment tool for FASD diagnosis. The MABC-2 total motor score was the most accurate using current guidelines, though its sensitivity was still low. Further investigation into inclusion of single subtests and/or using a less conservative cut-off in the Canadian Guideline is warranted.

## Background

Fetal alcohol spectrum disorder (FASD) is an umbrella term used to describe a combination of neurodevelopmental impairments and physical characteristics that result from prenatal alcohol exposure (PAE) [1]. The prevalence in Canada is estimated to be 0.79% of the population, although few Canadian prevalence studies have been conducted [2]. The severity of FASD varies with the frequency, timing, and amount of PAE [3, 4] and while PAE is a required criterion for the diagnosis of FASD, not all individuals with PAE meet criteria for an FASD diagnosis [5]. In addition to confirmed PAE, *Fetal alcohol spectrum disorder: a guideline for diagnosis across the lifespan* (Canadian Guideline) requires evidence of pervasive brain dysfunction defined by severe impairment in at least 3 of 10 neuro-developmental domains [5]. Diagnostic evaluation is completed by a multi-disciplinary team who conduct thorough developmental assessments, which include physical and neurological examination, to investigate pervasive brain dysfunction [5].

Occupational and/or physical therapists are often included on diagnostic teams for the purpose of assessing the motor domain which include gross motor, fine motor and visual-motor integration skills [5]. Gross motor skills use the large muscles of the body for balance, coordination, and strength to perform activities such as throwing, running and riding a bike. Fine motor skills use the small muscles of the hands for strength and dexterity to perform activities such as opening containers, drawing, and tying shoelaces. Visual motor skills use both the visual and motor systems combined (i.e., eye-hand coordination) to complete activities such as copying shapes and catching a ball. Difficulties with these motor skills can negatively impact day to day function (e.g. participation in gym, independence in dressing, and completing school work). It is well documented that motor skills are commonly impaired in children with PAE and FASD [3–9], yet this area is not always evaluated in assessment for FASD diagnosis.

The Canadian Guideline [5], lists a variety of motor assessment tools that are commonly used in FASD assessment for children aged 7–18 years including the Movement Assessment Battery for Children, Second edition (MABC-2) [10], the Beery-Buktenica Developmental Test of Visual-Motor Integration, Sixth Edition (BeeryVMI-6) [11], the Bruininks-Oseretsky Test of Motor Proficiency, Second Edition (BOT-2) [12] and the Rey Complex Figure Test (RCFT) [13]. *The Australian Guide to the diagnosis of FASD* (Australian Guide) lists the same assessment tools as the Canadian Guideline with the exception of the RCFT [13]. Other international FASD guidelines [14–16] did not include lists of motor assessment tools for use in FASD diagnosis. Research investigating sensitivity and specificity of assessment tools for use in FASD diagnosis is lacking.

The Canadian Guideline recommends using either total motor scores or multiple subtest scores from standardized motor assessments for confirmation of motor impairment [5]. However, it is unclear whether the use of total motor (fine and gross motor combined) or subtest scores (fine, gross and visual-motor separated) are more accurate. Recent literature suggests that fine and gross motor scores should be considered separately to support the diagnostic criteria for FASD, as they involve different neurodevelopmental areas and pathways [3, 4, 6]. Individuals with FASD present with heterogeneous impairments that may affect some areas of fine motor skills, gross motor skills, or both [3, 4, 6]. Recent literature supports the use of subtest scores in motor evaluation for FASD, to provide a more specific profile of motor impairments [17–19]. Further, complex gross and fine motor skills that involve multiple neural pathways are more likely to be impaired after PAE than basic motor skills [3, 6, 7, 20].

The Canadian Guideline uses 2 or more standard deviations below the mean (−2SD), as a cut-off to indicate a severe impairment in all of the neuro-developmental domains [5]. This cut-off is the standard for defining a severe deficit for many diagnoses, and is used in other scales and guidelines including the Diagnostic and Statistical Manual of Mental Disorders [21]. However, this cut-off is considered conservative and may not identify all children who have significant impairments. The -2SD cut-off was reviewed during the latest update of the Canadian Guideline. While a cut off of − 1.5 SD was considered, [5] it was decided that there was no empirical data to support the change [5] and there was concern about over-identification. Research in this area is needed to investigate which cut-off score is the most accurate (i.e., optimizing the balance between sensitivity and specificity).

Existing international FASD diagnostic guidelines [5, 13–16] vary considerably in regards to cut-off scores and use of subtests (Table 1). Cut off scores range from -2SD to −1SD (2nd to 16th percentile), indicating a wide variance in accepted impairment levels [5, 13–16]. Direct comparisons between these guidelines are difficult due to variability in the diagnostic criteria. The Australian Guide [13] and the Canadian Guideline [5] are unique in their use of subtests, though the terminology differs making direct comparison difficult.

**Table 1:** Comparison of FASD guideline features that relate to the neurodevelopmental assessment

Guideline	Diagnostic terms	Cut-off score to indicate a severe impairment	Total scores or subtests	Evidence of pervasive brain dysfunction
Canadian Guideline (2016) [5]	FASD with Sentinel Facial Features, FASD without Sentinel Facial Features	-2SD	Composite score or multiple subtest scores	Severe impairment in 3 or more of 10 neurodevelopment domains
Australian Guide (2016) [13]	FASD with 3 Sentinel Facial Features, FASD with < 3 Sentinel Facial Features	-2SD	Composite score or 1 or more major subdomain scores	Severe impairment in at least 3 neurodevelopmental domains
Updated Clinical Guidelines (2016) [15]	FAS, Partial FAS, ARND, ARBD	-1.5SD	Not specified	Impairment in at least 1 neurodevelopmental domain
University of Washington 4 Digit Code (2004) [14]	FAS, Partial FAS, ARND	-2SD	Not specified	Severe dysfunction in 3 or more domains of function
CDC Diagnostic Guidelines (2004) [16]	FAS	-1SD	Not specified	Deficit in 3 or more functional domains

Other factors not listed in this table contribute to the diagnoses listed above, such as confirmation of prenatal alcohol exposure, facial features, growth and structural abnormalities

Canadian Guideline = *Fetal alcohol spectrum disorder: a guideline for diagnosis across the lifespan,* Australian Guide = *Australian Guide to the diagnosis of Fetal Alcohol Spectrum Disorder (FASD),* Updated Clinical Guidelines = *Updated Clinical Guidelines for Diagnosing Fetal Alcohol Spectrum Disorders,* University of Washington 4 Digit Code = *Diagnostic Guide for Fetal Alcohol Spectrum Disorders: The 4-Digit Diagnostic Code, 3rd Edition,* CDC Diagnostic Guidelines = Center for Disease Control and Prevention’s *Fetal Alcohol Syndrome: Guidelines for Referral and Diagnosis, FASD* Fetal Alcohol Spectrum Disorder, *FAS* Fetal Alcohol Syndrome, *PFAS* Partial Fetal Alcohol Syndrome, *ARND* Alcohol-Related Neurodevelopmental Disorder, *ARBD* Alcohol-Related Birth Defects, *SD* Standard Deviation from the mean

In order to clarify diagnostic criteria regarding motor impairment in FASD, the following objectives of the study were identified:

1. Determine the diagnostic accuracy of motor assessment tools and subtests listed in the Canadian Guideline;
2. Determine if a severe motor impairment can be more accurately identified by using multiple subtest scores or total motor scores;
3. Investigate which cut-off is most accurate in identifying a motor domain impairment when assessing for FASD.

## Methods

### Study design

Cross-sectional diagnostic study using historical data obtained by patient file review. Ethics approval including a waiver of consent, was obtained from the University of Alberta Human Research Ethics Board.

### Participants

**Fig. 1:**
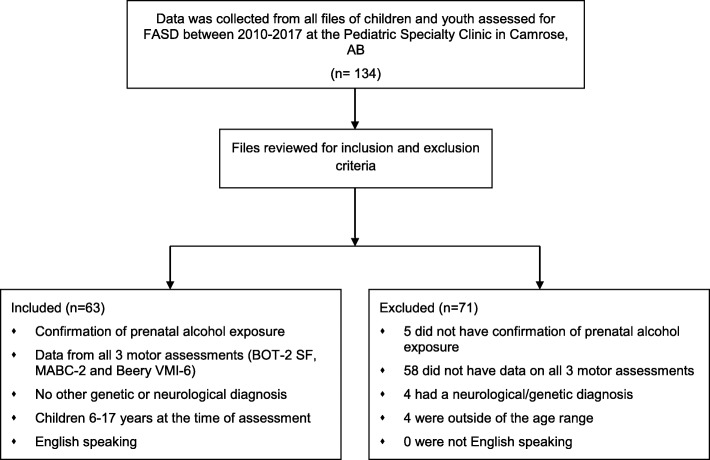
Review process of files

Children and youth had been assessed for FASD between 2010 and 2017 by the Alberta Health Services Camrose Pediatric Specialty Clinic, a diagnostic clinic that provides services in Alberta, Canada. The multi-disciplinary team includes a social worker, pediatrician, psychologist, speech language pathologist, occupational therapist and physical therapist. Medical files were eligible for inclusion if the children and youth were between 6 and 17 years at the time of the assessments and had confirmed PAE. PAE was confirmed in accordance with the Canadian Guideline by either: reliable clinical observation; self-report; reports by a reliable source; medical record documenting positive blood alcohol concentrations; alcohol treatment; or other social, legal or medical problems related to drinking during pregnancy [5]. All children included in this study met the PAE threshold suggested in the Canadian Guideline of 7 or more standard drinks per week or 2 episodes of 4 or more drinks on the same occasion (binge episodes) [5]. For this study, PAE was then categorized into High Risk (exposure to 7 or more drinks per week during all 3 trimesters) or Some Risk (7 or more drinks per week during 1 or 2 trimesters) and documented on the social worker intake form. Files were excluded if they did not include data from all three motor assessment tools, the MABC-2, the BeeryVMI-6 and the Bruininks-Oseretsky Test of Motor Proficiency, Second Edition, Short Form (BOT-2SF), or if they had received a genetic or other neurological diagnosis, which precluded a diagnosis of FASD. A sample size calculation indicated that a sample size of 52 was needed to calculate sensitivity and specificity with power of 0.8. Files of 134 children were initially reviewed, of which 71 did not meet inclusion criteria. A total of 63 files met criteria and were included in the analysis (Fig. 1).

### Diagnostic gold standard

The Canadian Guideline, the current gold standard for FASD diagnosis in Canada, states there must be evidence of impairment in 3 or more of the following neuro-developmental domains in addition to confirmed PAE: motor skills; neuroanatomy/neurophysiology; cognition; language; academic achievement; memory; attention; executive function; affect regulation; adaptive behaviour; social skills; or social communication [5]. The diagnostic criteria used in this study was evidence of impairment in 3 or more of the other non-motor neuro-developmental domains, resulting in an FASD diagnosis. Children with at least three severe impairments in non-motor domains were included to ensure that the gold standard for comparison was children who would have received a diagnosis without the inclusion of the motor assessment.

### Motor assessment tools

Motor impairment is considered significant for FASD diagnostic purposes if the total score or multiple subtest scores fall -2SD using a standardized motor assessment tool [5]. This study evaluated the MABC-2 (total score) and its three subtests, manual dexterity (MABC-2MD), aiming and catching (MABC-2 AC) and balance (MABC-2B), the BeeryVMI-6 and its Motor Coordination subtest (BeeryMC), and the BOT-2SF. These assessment tools are listed in the current Canadian Guideline, though it is not indicated whether the short form or complete form of the BOT-2 is preferred and whether the 2 supplemental tests of the BeeryVMI-6 should be completed [5]. The visual perception subtest of the BeeryVMI-6 (BeeryVP) was excluded because it does not include a motor component. The RCFT was excluded, as the results are not used for the motor domain in our clinic. Functional motor abilities were evaluated using a non-standardized activities of daily living interview with caregivers, obtaining information about eating, dressing, hygiene, chores, homework and leisure. Knowledge of FASD diagnosis was not known to the motor test administrators at the time of testing since the diagnostic decision was made after the completion of full neuropsychological testing.

The MABC-2, BeeryVMI-6 and the BOT-2 are all commonly and internationally used, standardized and norm-referenced assessment tools with well-established psychometric properties [10–12, 22–25]. These assessment tools have all been used in international research for motor assessment of children and youth with PAE and FASD [4, 7, 17–19, 26–30]. The MABC-2 is a motor assessment tool for children aged 3–17 years. It consists of 8 items divided into 3 subtests (manual dexterity, aiming and catching, and balance), which are combined to report a total motor score [10]. The BeeryVMI-6 assesses visual motor integration for individuals aged 2–100 years [11]. It consists of a developmental sequence of 30 geometric forms that are copied with paper and pencil. There are 2 optional supplemental tests, the BeeryVP and the BeeryMC, which evaluate visual and motor contributions to visual-motor integration separately. The BOT-2 assesses fine and gross motor function for individuals aged 4–21 years and is available in a complete form or a short form [12]. The complete form consists of 53-items that evaluate 8 areas of motor development including fine motor precision, fine motor integration, manual dexterity, upper limb coordination, bilateral coordination, balance, running speed and agility, and strength [12]. The short form consists of 14 items that were selected to represent abilities from the 8 subtests, to produce an overall motor proficiency score that is sufficiently reliable [12]. The manual indicates that the short form is a screening tool, yet some Canadian clinics are using it as an indication of significant motor impairment for the purposes of FASD diagnosis, largely due to time constraints.

### Statistical analysis

Data extraction was completed by members of the Pediatric Specialty Clinic. Data were de-identified, assigned a participant number and entered into a spreadsheet. Inter-rater agreement checks were completed on 10% of the files and agreement between raters was 98%. Discrepancies were discussed to reach consensus and inform ongoing data collection.

Descriptive statistics were calculated on all continuous (mean and standard deviation) and nominal variables (proportions). To determine diagnostic accuracy of the subtest and total motor scores, data from children with FASD and PAE without FASD were used. Sensitivity, specificity and overall diagnostic accuracy of the motor assessment tools were calculated using the McNemar χ2 statistic (a test used on paired nominal data) with 95% confidence intervals. A *p* value of < 0.05 was judged as statistically significant. IBM SPSSv23 (Armonk, New York) was used to conduct the analysis. Sensitivity and specificity are common statistical techniques for evaluating diagnostic accuracy. Sensitivity refers to the ability of a measure to correctly identify a condition in children who truly have that condition (true positive) and specificity refers to the ability of a measure to correctly rule out a condition in children who truly do not have the condition (true negative). Accuracy is the balance between optimal sensitivity and specificity. We made a priori decision that sensitivity values greater than 0.65 and specificity values greater than 0.75 were clinically useful for FASD assessment in our clinical context [31]. Since the diagnosis of FASD has substantial implications, high specificity is important to minimize over identification. Alteration of cut-off scores results in optimizing sensitivity or specificity at the expense of the other. The optimal cut-off will provide a balance of sensitivity and specificity considering implications for both under and over-identification. We analyzed cut-off scores at the 2nd, 5th, 9th and 16th percentiles to determine the optimal balance for motor assessment.

The prevalence of severe fine motor, gross motor and total motor impairments were calculated to determine the types and frequency of motor difficulties in our study. Fine motor prevalence was described as the proportion of children with a score of -2SD on the MABC-2MD, BeeryMC, or BeeryVMI-6. The gross motor prevalence was determined by the proportion of children with a score of -2SD on the MABC-2AC or the MABC-2B. Total motor prevalence was determined by a score of -2SD on the MABC-2 total motor score. The BOT-2SF did not contribute to the prevalence values, as only one child was identified as having a severe total motor impairment on the BOT-2SF and the MABC-2 identified this same child.

## Results

**Fig. 2:**
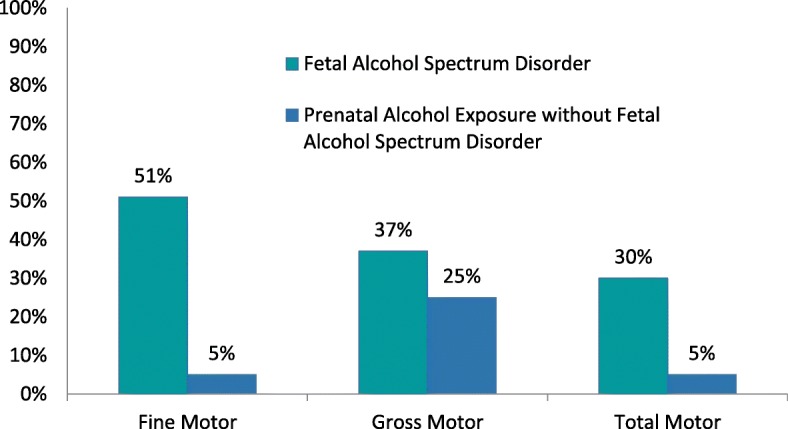
Prevalence of severe fine motor, gross motor and total motor impairments

Of the 63 children, 43 (68%) received an FASD diagnosis. The prevalence of severe gross, total and, in particular fine motor impairments, was higher in children with FASD compared to children who had confirmed PAE without FASD. Descriptive statistics for children and youth with FASD and PAE without FASD are presented in Table 2 and Fig. 2.

**Table 2:** Descriptive statistics (*n* = 63)

	FASD (*n* = 43)	PAE without FASD (*n* = 20)
Sex female/male	10/33	11/9
Mean age, years (SD)	10 years, 6 months (2.79)	10 years, 4 months (2.97)
Amount of PAE (High/Some)	12%/88%	15%/85%
Mean IQ score (SD)	77.23 (12.65)	88.68 (8.38)
Mean IQ percentile (SD)	12.24 (15.08)	24.15 (18.11)
Comorbidities		
ADHD	81%	50%
ODD	2%	5%
Learning Disabilities	35%	35%
Other (e.g. DCD, mental health conditions, medical conditions)	60%	40%
Difficulties with ADLs^a^		
Eating	35%	42%
Dressing	54%	32%
Shoelace Tying	76%	59%
Bathing	35%	28%
Personal Hygiene	36%	42%
Toileting	31%	53%
Bike Riding	30%	43%

^a^sample sizes varied for the ADL tasks: FASD *n* = 33–42, PAE *n* = 14–19

*FASD* Fetal Alcohol Spectrum Disorder, *PAE* Prenatal Alcohol Exposure, *SD* Standard Deviation, *IQ* Intellectual Quotient, *ADHD* Attention Deficit Hyperactivity Disorder, *ODD* Oppositional Defiance Disorder, *DCD* Developmental Coordination Disorder, *ADLs* Activities of Daily Living (collected via a non-standardized caregiver interview).

### Diagnostic accuracy of motor assessment tools

Sensitivity and specificity of the motor assessments were calculated at −2SD (Table 3). The sensitivity of the BOT-2SF was extremely low (0.02; 95% CI 0.00–0.12). The MABC-2 total motor score was statistically more sensitive than the BOT-2SF, though still low and not considered clinically useful (0.30; 95% CI 0.17–0.46; *p* < 0.01). The MABC-2 total motor scores indicating a higher prevalence of severe motor difficulty, is consistent with the prevalence of functional concerns observed clinically and reported by caregivers (Table 2). Sensitivity and specificity were also examined for subtests and compared to total motor scores. The total motor score of the MABC-2 was more sensitive than any combination of multiple subtest scores. The BeeryMC subtest was found to have the highest sensitivity (0.38; 95% CI 0.23-0.54). However, when compared to the sensitivity of the total motor score of the MABC-2 (0.30) the difference was not statistically significant (*p* = 0.61). No combination of subtests and functional tasks were found to be more accurate than the total score of the MABC-2.

**Table 3:** Sensitivity and Specificity of the Motor Assessment Tools (*n* = 63) at − 2 SD

Assessment Tool	Sensitivity	Specificity
BOT-2SF	0.02	1.00
BeeryVMI-6	0.16	1.00
BeeryMC	0.38	0.95
MABC-2 Total	0.30	0.95
MABC-2MD	0.23	1.00
MABC-2 AC	0.19	0.80
MABC-2B	0.23	0.90

### Exploratory analysis of alternate percentile cut-offs

Using the recommended -2SD (2nd percentile) cut off score of the current Canadian Guideline, the highest sensitivity obtained on listed motor assessment tools was 0.30. An exploratory analysis was completed using cut-offs at the 5th, 9th and 16th percentiles to determine the optimal balance between sensitivity and specificity for motor assessment tools (Table 4). The most accurate cut-off for the total motor score of the MABC-2 was found to be the 2nd percentile (0.30; 95% CI 0.17–0.46), as the specificity dropped too low at the alternate percentile cut offs. When using multiple subtests, accuracy was greatest when combining the BeeryMC and the MABC-2MD at the 5th percentile (sensitivity 0.40, specificity 1.00). When using a single subtest, the highest accuracies were found for the BeeryMC at the 5th percentile (0.68/0.90) and at the 9th percentile (0.75/0.84). Administration of both the MABC-2MD and the BeeryMC (then using the results of either subtest score), also resulted in high accuracies at the 5th percentile (0.75/0.84) and the 9th percentile (0.85/0.79). Both of these options resulted in substantially higher sensitivities than those obtained using the current recommended criterion for motor assessment in the Canadian Guideline, while retaining high specificities.

**Table 4:** Assessment Accuracy at Various Cut-Off Percentiles for the Motor Assessment Tools

Sensitivity and Specificity at Various Cut-Off Percentiles (*n* = 63)				
Percentile (SD)	2nd (−2SD)	5th (− 1.5SD)	9th ^a^	16th (−1SD)
BOT-2SF	0.02/1.00	0.09/0.95	0.35/0.85	0.61/0.55
BeeryVMI-6	0.16/1.00	0.30/0.90	0.44/0.80	0.63/0.70
BeeryMC	0.38/0.95	**0.68/0.90**	**0.75/0.84**	0.83/0.63
MABC-2 Total	0.30/0.95	0.33/0.85	0.63/0.65	0.72/0.45
MABC-2MD	0.23/1.00	0.47/0.95	0.61/0.85	0.67/0.70
MABC-2 AC	0.19/0.80	0.33/0.75	0.39/0.75	0.44/0.60
MABC-2B	0.23/0.90	0.37/0.85	0.49/0.60	0.58/0.55
MABC-2MD *and* BeeryMC	–	0.40/1.00	–	–
MABC-2B *and* MABC-2 AC	–	0.20/0.90	–	–
MABC-2MD *and* MABC-2B	–	0.23/1.00	–	–
MABC-2B *and* BeeryMC	–	0.33/1.00	–	–
MABC-2MD *or* BeeryMC	–	**0.75/0.84**	**0.85/0.79**	–
MABC-2B *or* MABC-2 AC	–	0.50/0.68	–	–
MABC-2MD *or* MABC-2B	–	0.63/0.79	–	–
MABC-2B *or* BeeryMC	–	0.73/0.74	–	–

^a^does not correspond with a standard deviation cut-off

Dashes (−) indicate not tested in exploratory analyses

Bolded values indicate optimal balance between sensitivity and specificity

*SD* Standard Deviation, *BOT-2SF* Bruininks-Oseretsky Test of Motor Proficiency, Second Edition, Short Form, *BeeryVMI-6* Beery-Buktenica Developmental Test of Visual-Motor Integration, Sixth Edition, *BeeryMC* Beery-Buktenica Developmental Test of Visual Motor Integration 6th edition Motor Coordination subtest, *MABC-2 Total* Movement Assessment Battery for Children, Second edition, Total, *MABC-2MD* Movement Assessment Battery for Children 2nd edition, Manual Dexterity subtest, *MABC-2 AC* Movement Assessment Battery for Children 2nd edition, Aiming and Catching subtest, *MABC-2B* Movement Assessment Battery for Children 2nd edition, Balance subtest

## Discussion

The Canadian Guideline for diagnosis of FASD lists the BOT-2, MABC-2 and BeeryVMI-6 for motor assessment in children with suspected FASD [5]. The findings of this study indicate the BOT-2SF is not an accurate assessment tool for evaluating motor impairment in this population, identifying only 2% of children with FASD as having a severe motor impairment. Since 2% is the prevalence expected in the general population, one would expect the rate would be higher among children and youth with FASD. We suggest that use of the BOT-2SF in FASD diagnostic assessment should be reconsidered. Appropriateness of the complete and short forms should be considered separately, as our study did not investigate the BOT-2 complete form. There is some evidence that the complete form is able to detect motor impairments in this population as it was previously found to identify 9.5% of children with FASD as having a severe motor impairment [4]. The BeeryVMI-6 identified 16% of children with FASD with a severe motor impairment, suggesting it has clinical value. We found the MABC-2 to have the highest accuracy, identifying 30% of children with FASD as having a severe motor impairment. It may have identified more children because it assesses more complex motor skills (e.g., constructing a triangle using nuts and bolts and hopping on one leg in a specific pattern) which require coordination of multiple motor sub-systems [7]. The literature suggests that complex motor skills are more often affected than basic motor skills in individuals with FASD [6, 7].

The Canadian Guideline recommends the use of total motor or multiple subtest scores at − 2 SD to provide evidence of a severe motor impairment [5], resulting in a more conservative diagnostic criteria compared to other guidelines [15, 16]. The BeeryMC subtest was found to have the highest sensitivity at -2SD (0.38), which supports its use in FASD diagnostic assessment. Our findings are in line with other research which also detected high levels of motor impairment in this population using the BeeryMC subtest [17]. Previous research found the -2SD cut-off to be too restrictive and evaluated prevalence of motor impairment at -1SD (16th percentile cut-off) in children with FASD [17–19]. Our results demonstrated that although the total motor score of the MABC-2 had the highest accuracy using current recommendations, this value is still low. Our results suggest that diagnostic accuracy for the motor domain is improved when using the cut-off score of − 1.5 SD, particularly using the BeeryMC subtest. This altered criterion resulted in correctly identifying more children as having a motor impairment without increased false identification, resulting in overall greater accuracy compared to current guideline recommendations. This finding highlights that the recommendations for motor assessment in the current Canadian Guideline do not have sufficient statistical accuracy to identify motor impairment. Our results demonstrated that while sensitivity increased further at -1SD (16th percentile), optimal balance with specificity was not attained which could result in over-identification. Further investigation of the inclusion of single subtests and/or use of a -1.5 SD cut-off level in the Canadian Guideline is warranted to confirm these findings.

Prevalence rates of fine and gross motor deficits among children with FASD and PAE support that motor skills should regularly be assessed when considering an FASD diagnosis. In a meta-analysis of children with moderate to high PAE, gross motor skills were found to be 2.9 times more likely to be impaired [3] and significant fine motor impairments are also reported in children with PAE [6, 8, 9]. Our findings of the prevalence of both fine and gross motor impairments and functional difficulties found in children with PAE and FASD were consistent with these studies. Involvement of both occupational therapy and physical therapy is warranted as part of a multi-disciplinary team to provide input towards diagnosis and recommendations in FASD diagnostic clinics.

### Limitations

This study had several limitations. PAE was reported by mothers retrospectively, which may have led to recall bias. However, PAE was also confirmed, when possible, by other reliable sources as listed in the Canadian Guideline. In addition, activity of daily living abilities were based on parental report and clinician observation, and not a standardized, norm-referenced assessment tool. Clinicians were not masked to PAE, as all children in our study had PAE (i.e. they are referred to our clinic when FASD is suspected due to PAE). However, knowledge of FASD diagnosis was unknown at the time of assessment.

## Conclusions

Our results suggest that the BOT-2SF is an inaccurate assessment tool for identifying a motor impairment in this population and therefore its use in FASD assessment should be reconsidered. The total motor score of the MABC-2 was more accurate than: the BOT-2SF, use of multiple subtest scores from the MABC-2, or the BeeryVMI-6. Further investigation into inclusion of single subtests and/or using a -1.5 SD cut-off level in the Canadian Guideline is warranted. The findings of this study support and clarify the Canadian Guideline potentially leading to more accurate diagnosis of FASD.

## Abbreviations

Australian Guide: The Australian Guideline to the diagnosis of FASD
BeeryMC: Beery-Buktenica Developmental Test of Visual Motor Integration 6th edition Motor Coordination subtest
BeeryVMI-6: Beery-Buktenica Developmental Test of Visual Motor Integration 6th edition
BeeryVP: Beery-Buktenica Developmental Test of Visual Motor Integration 6th edition Visual Perception subtest
BOT-2: Bruininks-Oseretsky Test of Motor Proficiency 2nd edition
BOT-2SF: Bruininks-Oseretsky Test of Motor Proficiency 2nd edition Short Form
Canadian Guideline: Canadian FASD diagnostic guideline, Fetal alcohol spectrum disorder: a guideline for diagnosis across the lifespan
CDC Diagnostic Guidelines: Center for Disease Control and Prevention’s Fetal Alcohol Syndrome: Guidelines for Referral and Diagnosis
FASD: Fetal Alcohol Spectrum Disorder
MABC-2: Movement Assessment Battery for Children 2nd edition
MABC-2 AC: Movement Assessment Battery for Children 2nd edition, Aiming and Catching subtest
MABC-2B: Movement Assessment Battery for Children 2nd edition, Balance subtest
MABC-2MD: Movement Assessment Battery for Children 2nd edition, Manual Dexterity subtest
PAE: Prenatal Alcohol Exposure
RCFT: Rey Complex Figure Test
SD: Standard Deviations from the mean
University of Washington 4 Digit Code: Diagnostic Guide for Fetal Alcohol Spectrum Disorders: The 4-Digit Diagnostic Code, 3rd Edition
Updated Clinical Guidelines: Updated Clinical Guidelines for Diagnosing Fetal Alcohol Spectrum Disorders
